# Guided Wave Characteristic Research and Probabilistic Crack Evaluation in Complex Multi-Layer Stringer Splice Joint Structure

**DOI:** 10.3390/s23229224

**Published:** 2023-11-16

**Authors:** Jian Chen, Yusen Xu, Shenfang Yuan, Zhen Qin

**Affiliations:** Research Center of Structural Health Monitoring and Prognosis, State Key Laboratory of Mechanics and Control for Aerospace Structures, Nanjing University of Aeronautics and Astronautics, 29 Yudao Street, Nanjing 210016, China; cj1108@nuaa.edu.cn (J.C.); xuyusen@nuaa.edu.cn (Y.X.); zhenqin@nuaa.edu.cn (Z.Q.)

**Keywords:** multi-layer, multi-rivet, guided wave, probabilistic mining diagnosis, path wave band feature

## Abstract

Multi-layer and multi-rivet connection structures are critical components in the structural integrity of a commercial aircraft, in which elements like skin, splice plate, strengthen patch, and stringer are fastened together layer by layer with multiple rows of rivets for assembling the fuselage and wings. Their non-detachability and inaccessibility pose significant challenges for assessing their health states. Guided wave-based structural health monitoring (SHM) has shown great potential for on-line damage monitoring in hidden structural elements. However, the multi-layer and multi-rivet features introduce complex boundary conditions for guided wave propagation and sensor layouts. Few studies have discussed the guided wave characteristic and damage diagnosis in multi-layer and multi-rivet connection structures. This paper comprehensively researches guided wave propagation characteristics in the multi-layer stringer splice joint (MLSSJ) structure through experiments and numerical simulations for the first time, consequently developing sensor layout rules for such complex structures. Moreover, a Gaussian process (GP)-based probabilistic mining diagnosis method with path-wave band features is proposed. Experiments on a batch of MLSSJ specimens are performed for validation, in which increasing crack lengths are set in each specimen. The results indicate the effectiveness of the proposed probabilistic evaluation method. The maximum root mean squared error of the GP quantitative diagnosis is 1.5 mm.

## 1. Introduction

In civil aviation, the fuselage and wings of a commercial aircraft are typically composed of numerous individual structural components, which are manufactured separately and then assembled through connections like riveting and bolting [1]. The connecting parts of these components are critical regions susceptible to fatigue crack damages due to stress concentration at fastening holes under intricate service conditions. However, it is difficult to examine their structural state since they are usually non-disassembled and located in hard-to-reach regions. In recent decades, the aviation industry has sought structural health monitoring (SHM) techniques for monitoring and managing the aircraft structure. Different SHM methods have been developed for real-time structural state and damage monitoring [2,3,4,5,6,7]. Among them, the guided wave-based method has been deemed as one of the promising techniques. Its basic idea is to use the piezoelectric transducer (PZT) to excite the guided wave propagating spreading around the structure [8]. The occurrence and the growth of the damage are identified by interpreting feature changes of guided wave signals acquired with PZT sensors. Therefore, this technique has the capability of monitoring a region and accessibility of hidden structural elements. It is of great potential to employ the guided wave (GW)-based SHM for crack damage monitoring in aircraft’s connecting structures.

Some preliminary studies were conducted to monitor the crack presence and growth in the connection structures with the GW-based SHM [9,10]. Ihn and Chang et al. [11] monitored the crack growth in a riveted lap joint structure using a PZT sensor/actuator network, which is composed of two aluminum plates fastened with rivets. The damage index from the damaged guided wave signal and its baseline was extracted to indicate the growth of the crack. He et al. [12,13] used guided wave changes in amplitude and phase to represent crack growth in the rivet hole of a lap joint structure composed of two plates. Stolze and Worden et al. [14] used PZT sensor layers for damage monitoring in the riveted strap joint of two aluminum plates. The amplitude characteristic of the guided wave was used to denote crack presence at rivet holes. Asadi and Aliabadi et al. [15] developed a baseline-free guided wave technique to detect crack initiation at bolt holes of a plate structure mounted with a stringer. Chen et al. [16] proposed a framework for fatigue crack assessment based on guided wave-convolutional neural network (GW-CNN) integration and differential wavelet spectroscopy, where lapped structures were tested. In addition, guided wave imaging methods were also adopted to visualize the location of fatigue cracks in bolted lap joint structures with PZT arrays. Lissenden et al. [17] used a tomography algorithm to detect and locate the crack in an aluminum plate with five open fastener holes. Quaegebeur et al. [18] developed a correlation-based imaging technique to locate the crack damage in the two plate structures fastened with a row of rivets. Wandowski et al. [19] and Bae et al. [20] adopted the scanning laser doppler vibrometer to acquire guided wave fields to determine possible damaged areas in the connection area. All these studies have shown the effectiveness of the GW-based SHM on crack damage monitoring in aircraft connecting structures.

However, the above studies mainly involved simplified structures like plates with holes or two fastened plates. Few studies discuss more realistic problems where multi-layer elements, like skin, splice plate, repair patch, and stringer, are fastened together layer by layer with multiple rows of rivets from different directions. This kind of complex multi-layer and multi-rivet connection structure brings complex boundary conditions for guided wave propagation [21]. Reflection, transmission, and mode conversion occur when the guided wave meets the structural boundaries, fastening holes, rivets, and bolts, resulting in amplitude attenuation and wave packet aliasing. Damage information in the guided wave signal will be masked, increasing the difficulties for reliable damage diagnosis [22]. Moreover, due to the interference from the multi-layer structural elements and multiple rows of rivets, there is limited space for arranging the PZT sensors. There is an urgent need for studies on the guided wave propagation characteristics in such complex multi-layer and multi-rivet connection structures for reliable damage diagnosis.

In addition, most studies mentioned above mainly discuss the detection and localization problem, and few of them studied the quantification of the crack size in the complex multi-layer and multi-rivet connection structure. Crack quantification means constructing a diagnosis model mapping the guided wave feature to the crack size, based on which the size of the crack in the structure can be evaluated. Usually, this is accomplished by conducting monitoring tests of a batch of identical structures and training the model like polynomial [23,24] and neural network [25,26] as the diagnostic model. There are more difficulties in crack quantification for the complex multi-layer and multi-rivet connection structure. Firstly, producing a clear wave packet of the A0 or S0 mode of the guided wave for damage feature extraction is difficult, which is commonly adopted in existing studies [27]. In addition, because of the inherent uncertainties during structure manufacturing and service conditions, guided wave signals in the batch of complex multi-layer and multi-rivet connection structures are quite different. Guided wave features are probabilistically distributed, even from the bath of identical structures. More efforts on feature extraction and its uncertainty consideration are needed for the crack evaluation in complex multi-layer and multi-rivet connection structures.

The primary objective of this paper is to develop a reliable crack evaluation method using guided waves for complex multi-layer and multi-rivet connection structures in civil aircraft, addressing the challenges associated with the structural integrity assessment of multi-layer and multi-rivet connection structures. To achieve this, this paper performs comprehensive research on guided wave propagation characteristics in the multi-layer stringer splice joint (MLSSJ) structure for the first time. This structure is the typical connection area of the skin, stringer, and frame of the aircraft fuselage in a commercial aircraft. The guided wave in the skin affected by the stringer, guided wave through stringers and joints, guided wave affected by simulated damages, as well as numerical simulation are studied. Based on these studies, PZT layout rules for such complex structures are proposed for crack monitoring, considering the crack sensitivity and space limitation. Moreover, a Gaussian process (GP)-based probabilistic mining diagnosis method with path-wave band features is proposed. The signal of reflection and scattering in the propagation path of the guided wave are combined for evaluating the damage index of the guided wave signal. Then, the GP-based method is used for modeling the distribution of the damage index for crack diagnosis. Finally, a batch of MLSSJ structures with machined cracks are used for the validation of the proposed method. Through these studies, information about how the guided wave is affected by multi-layer and multi-rivet connections can be provided, as well as the effects of damages, instructing sensor deployment on such kinds of structures and the development of reliable crack diagnosis methods. In addition, the proposed GP-based probabilistic mining diagnosis method provides a useful damage diagnosis tool for crack evaluation during ground tests of full-scale aircraft or on-line monitoring of aircraft.

This paper is organized as follows. Section 2 introduces the complex multi-layer stringer splice joint structure and studies its guided wave propagation characteristics. Section 3 introduces GP-based probabilistic mining diagnosis with path-wave band features. In Section 4, a crack evaluation of the MLSSJ structures is carried out. The conclusion is made in Section 5.

## 2. Guided Wave Characteristics Investigation in MLSSJ Structure

The basics of the GW-based SHM are to extract changes of the guided wave propagating in the structure and perform damage diagnosis with these changes. Therefore, research on guided wave propagation characteristics in the MLSSJ structure is important.

### 2.1. Complex Muzlti-Layer Stringer Splice Joint Structure

**Figure 1:**
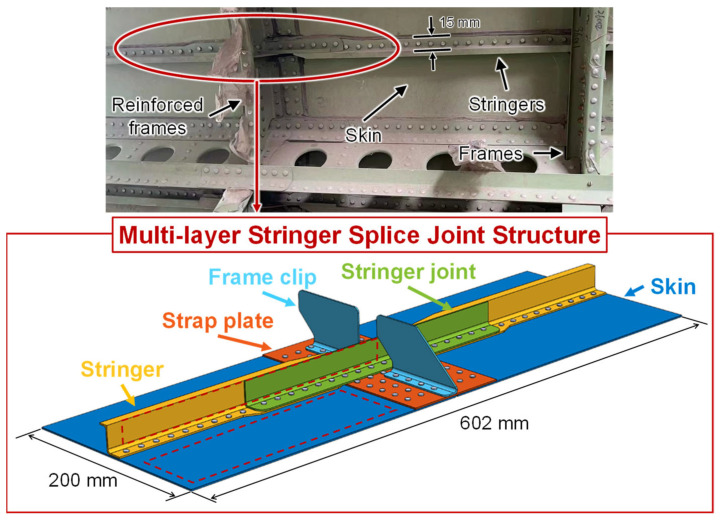
Geometries of the complex multi-layer stringer splice joint structure.

Figure 1 shows the MLSSJ structure, which is designed to emulate the critical longitudinal connection part of the fuselage. The two fuselage segments’ skin, stringer, and frame are connected and strengthened by the strap plate and stringer joint. Its dimensions are designed with reference to the real structure of a commercial aircraft. The thickness of the skin is 1.5 mm, while the one in the real structure is 1.6 mm. The height of the stringer is 25.5 mm, while the one in the real structure is 24 mm. Also, some simplifications are made, such as the frame, and the curvatures of the skin are not considered. These elements are fastened with multiple rivets layer by layer. The structure is made with the 2024 aluminum alloy and its whole size is determined by considering the experiment capability and costs.

### 2.2. Guided Wave Propagation Characteristics in Multi-Layer Components

**Figure 2:**
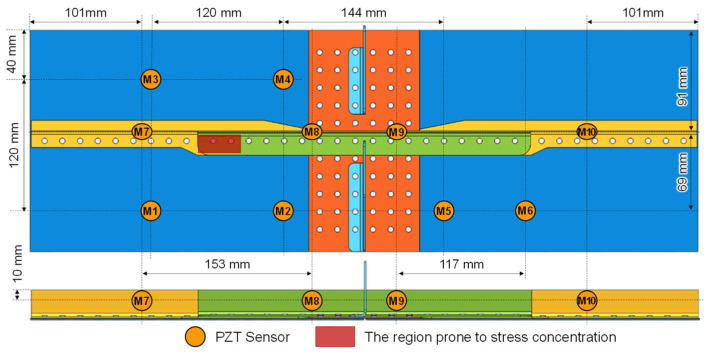
PZT arrangement for research of guided wave propagation characteristics.

According to the reference [16,28], the stringers usually undergo cyclic stretch load. Therefore, the stress concentration is more likely to occur near the rivet holes of the stringer joint as shown in Figure 2. These stress concentrations may lead to fatigue crack initiation, which are critical regions to be monitored. For these regions, there is limited space for the arrangement of the PZTs due to the existence of rivets and complex structure geometries. As shown in Figure 1, the first possible space is the inner surface of the skin. And the second possible space is the web of the stringer. Therefore, assuming the crack may occur at the stringer joints, the positions of M1, M2, M3, and M4 are determined for monitoring the region in the skin, as shown in Figure 2. And M7 and M8 are used for monitoring the region in the stringer. The distance of these PZTs is chosen according to the propagation velocity of the S0 and A0 modes in the plate structure with 1.5 mm so that the S0 and A0 modes are clear in such kind of structure. Additionally, considering the need to investigate the guided wave propagation characteristics of the MLSSJ, it is essential to arrange various types of channels for guided wave signal acquisition. Therefore, the M9, M10, M5, and M6 are deployed. It should be noted that PZTs M7, M8, M9, and M10 are positioned on the vertical web of the stringer.

**Figure 3:**
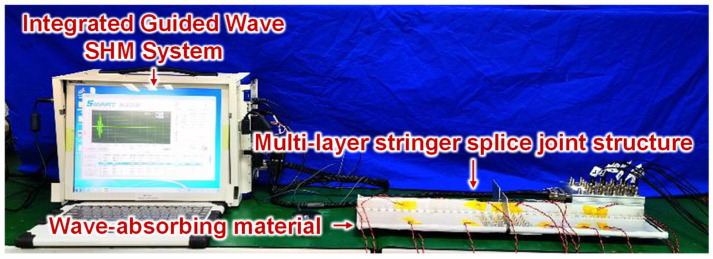
Experimental setup for guided wave propagation characteristics study.

The integrated guided wave SHM system developed by the authors’ group [29] is used to excite and collect guided wave signals. The signal excitation waveform is a three-cycle Hanning-windowed tone burst with an amplitude of 70 V. The sample rate of the guided wave signal is set as 30 MHz. In this experiment, the structure is fastened with countersunk bolts rather than rivets so that the guided wave propagation with or without specific structural elements can be revealed. As mentioned above, the MLSSJ structure is a truncation of the real structure, with the skin and the stringer being truncated compared to a complete structure. This truncation may introduce additional reflections of the guided waves compared to the real structure. To reduce the effect of the reflection, a wave-absorbing material is applied around the structure during structural experiments, as shown in Figure 3.

For studying the guided wave propagation characteristics, the central frequency of the excitation waveform is tested preliminarily. The guided wave in the plate structure usually has many propagation modes depending on the central frequency of the excitation wave. The more wave modes there are, the more complex the guided wave signal is. Therefore, in the guided wave-based SHM, usually the frequency region, such as bellowing 250 kHz is chosen for a 1.5 mm thick plate, so that only the A0 mode and the S0 mode are excited in the structure [2,8,30]. In addition, the temporal width of the wave packet relies on the central frequency. The lower the frequency is, the wider the wave packet is. To avoid the temporal width of the wave packet being too large, leading to the mix of the A0 mode to the reflection and scattering, a frequency larger than 50 kHz is adopted. Therefore, the central frequency is tried from 50 kHz to 250 kHz, with an interval of 10 kHz.

**Figure 4:**
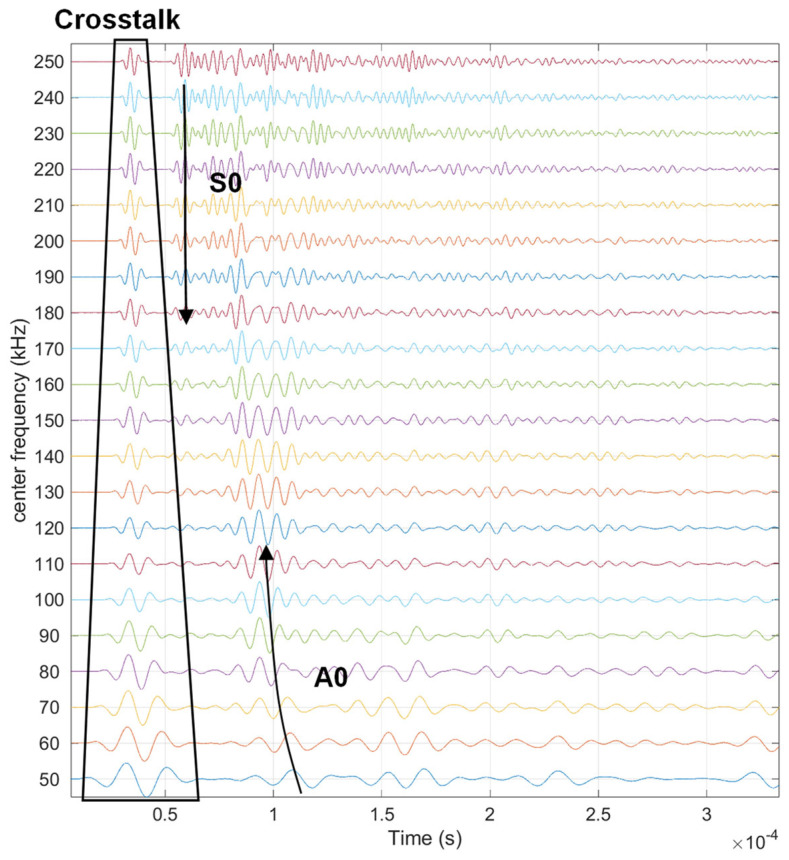
Guided wave signals at different frequencies.

Typical guided wave signals at different frequencies are shown in Figure 4. The frequency mainly affects the wave mode excited in the structure, as well as the temporal width of the wave packet, consequently affecting the mixing of the direct wave, reflection, and scattering. Selecting appropriate frequencies can have a clear wave packet for analysis. Therefore, in the experimental analysis and numerical analysis, two typical frequencies, 60 kHz and 230 kHz, are selected so that the A0 mode and S0 mode are clear.

#### 2.2.1. Guided Wave in the Skin Affected by the Stringer

**Figure 5:**
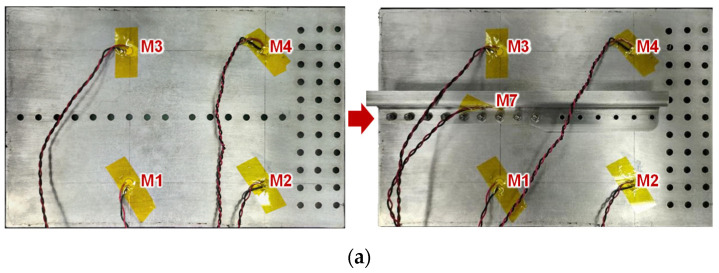

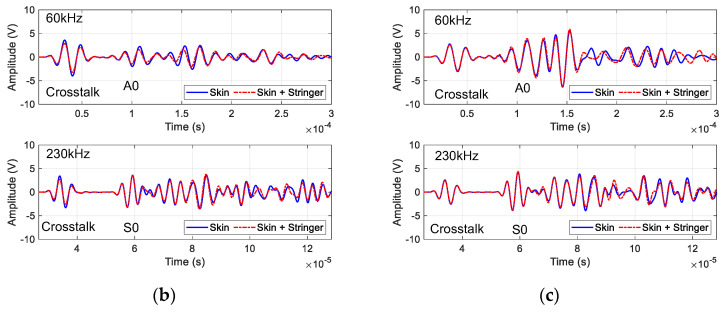
Guided wave signal from typical channels: (**a**) experimental setup; (**b**) signal of channel M1–M3; (**c**) signal of channel M2–M4.

To discuss the effects of the stringer, the stringer is removed and assembled on the skin. Typical guided wave signals from channels M1–M3 and M2–M4 are shown in Figure 5. Here, channel M1–M3 means M1 is the actuator and M3 is the sensor. The crosstalk is a useless interface signal introduced by circuits. These channels denote the guided wave propagation in the skin. It can be found that, after assembling the stringer, the guided wave signal changes in the amplitude and waveform. However, the change is small, especially for the wave packet of the S0 mode. Furthermore, the wave energy reduction is evaluated for channels M1–M2, M1–M3, M1–M4, M2–M3, and M2–M4, which are 10%, 2%, 4%, 10%, and 5%, respectively. The energy reduction is also small, showing that the guided wave is easier to propagate in its single-layer waveguide. The signal excited in the skin is difficult to propagate from the skin to the stringer.

#### 2.2.2. Guided Wave Propagation through Stringers and Joints

**Figure 6:**
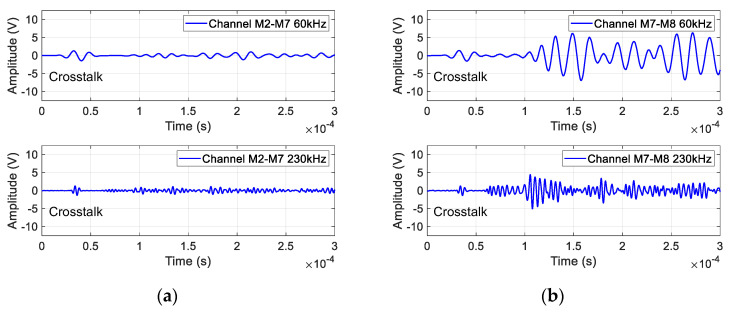
Typical GW signal of channel: (**a**) M2–M7; (**b**) M7–M8.

Figure 6a illustrates the guided wave signals from channel M2–M7. It can be observed that only a small portion of the guided wave excited on the skin can propagate to the stringer, and the mode of the guided wave signal also changes. This is due to the inefficient coupling between the skin and the stringer, as well as the right-angle bend between the stringer web and the stringer edge. Therefore, PZTs may be deployed on the stringer to monitor its crack damage.

Figure 6b illustrates the guided wave signals from channel M7–M8. The PZTs M7 and M8 are arranged on the webs of the stringer and stringer joint. The narrow and limited guided wave propagation space introduces more boundary reflection. The energy in the stringer is more focused than that in the skin. Moreover, the webs of the stringer and the stringer joint are contacted, by which more energy can be transferred. Therefore, the amplitude of the signal received by M8 is large, but it is difficult to find a clear wave packet of the S0 mode or the A0 modes because of the superposition of boundary reflections, mode conversion, as well as scattering of the rivet holes.

**Figure 7:**
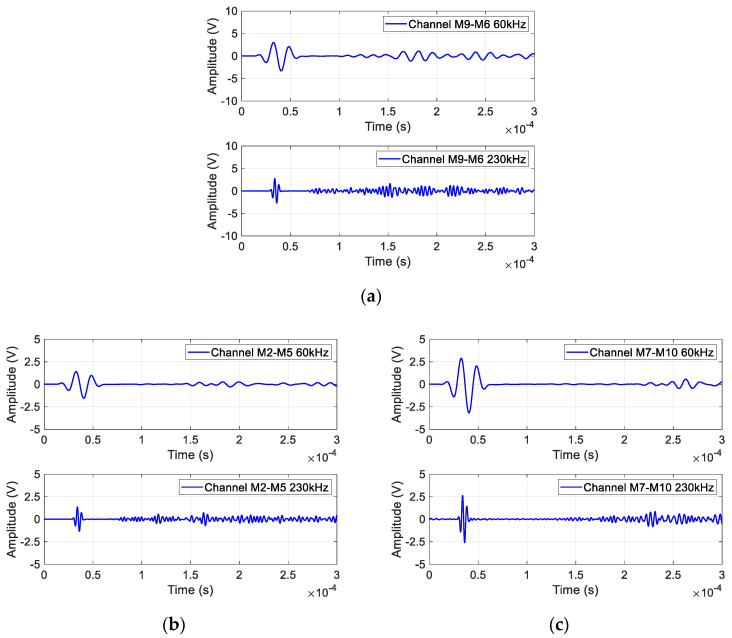
Typical GW signal of channel: (**a**) M6–M9; (**b**) M2–M5; (**c**) M7–M10.

In addition, there are situations in which guided waves propagate through three-layer waveguides in the complex MLSSJ structure. In Figure 7a, the guided wave signal of channel M6–M9 is given, where the wave is excited on the skin, and the sensor is arranged on the stringer joint. This channel shares some similarities with channel M2–M7, but a notable distinction lies in the guided wave’s traversal of an additional waveguide layer. During the propagation of the guided wave signal, multiple mode conversions occur, particularly pronounced at 230 kHz, resulting in the superposition of different wave modes. This phenomenon leads to a significant guided wave amplitude attenuation, resulting in a small signal when passing through three layers.

Figure 7b and Figure 6c illustrate the guided wave signal of M2–M5 and M7–M10. In channel M2–M5, the actuator M2 and sensor M5 are distributed on two different skins, connected by the strap plate and the stringer joint. Similarly, in channel M7–M10, the actuator and sensor are distributed on two different stringers as is demonstrated in Figure 2. The guided wave signals in these channels exhibit lower amplitudes and undergo multiple mode conversions. Notably, the M7–M10 channel, covering an extended distance, necessitates a prolonged duration for the direct wave to propagate. Consequently, the guided wave signal in this channel emerges much later, specifically around 2.5 × 10^−4^ s at 60 kHz, showcasing the delayed appearance of the direct wave in Figure 7c.

In conclusion, these results indicate that utilizing such a propagating path for damage monitoring is insufficient.

#### 2.2.3. Numerical Simulation of Guided Waves in the MLSSJ Structure

Finite elements simulation of the guided wave propagation is also conducted based on the COMSOL Multiphysics computational platform [31]. The geometric model of the structure is created as the same as the real structure. The low-reflecting boundary condition is adopted to minimize the reflection of waves at the boundaries. Two simulations are carried out, in which PZTs M2 and M7 are used as the actuator, respectively. The excitation waveform is chosen as the one with the central frequency of 230 kHz, consistent with the experiment.

**Figure 8:**
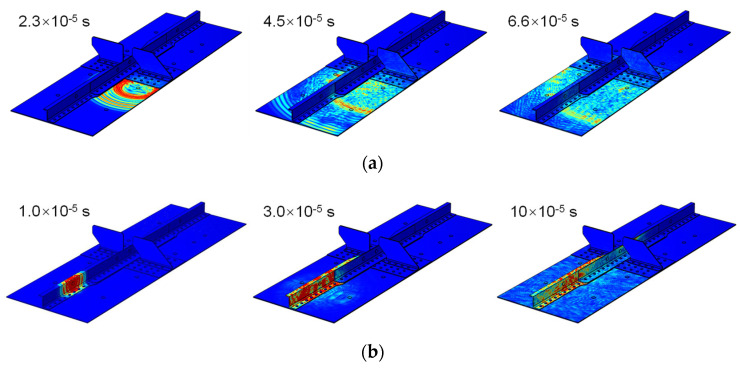
Guided wave simulation and wave field at different time instants: (**a**) M2 as the actuator; (**b**) M7 as the actuator.

In Figure 8a, the guided wave propagation field is given at three different time instants. The guided wave propagates well in the skin. However, when the wave propagates to the other structural elements, like the stringer or the strap plate, amplitude attenuation is found. There is limited guided wave propagating to the right skin. From Figure 8b, the boundary reflection in the stringer and stringer superimposes each other due to the narrow waveguides of the stringer. Only a small portion of the guided wave passes to the stringer joint at 3 × 10^−5^ s, where the S0 mode propagates in front of the A0 mode. But, after a period of propagation, a portion of guided waves transferred to the stringer joint, as the superposition of boundary reflections, mode conversion, and scattering is large. The simulation is consistent with the experimental result.

### 2.3. Guided Wave Affected by Simulated Damages in MLSSJ Structure

**Figure 9:**
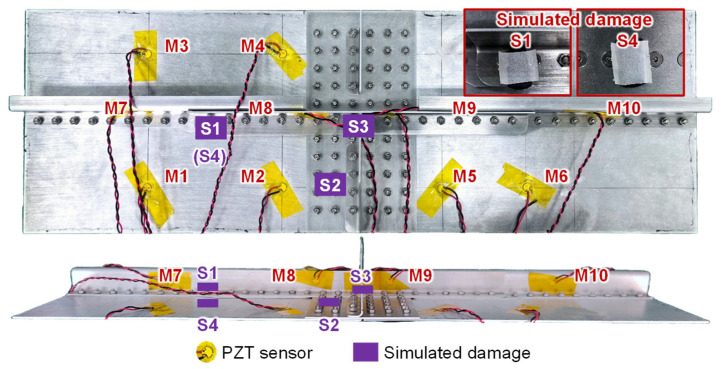
Simulated damage placed on the complex MLSSJ structure.

Furthermore, the effects of simulated damage on guided wave propagation are studied. The experiment setup of the simulated damage is given in Figure 9. The damage is set by the wave-absorbing material, which can change the local stiffness of the structure like the real damage. Before applying the simulated damage, the baseline signals are collected from each channel. The guided wave signals are excited and collected after adding simulated damages, respectively. Simulated damages are applied to four locations separately, named S1, S2, S3, and S4, as shown in Figure 9.

**Figure 10:**
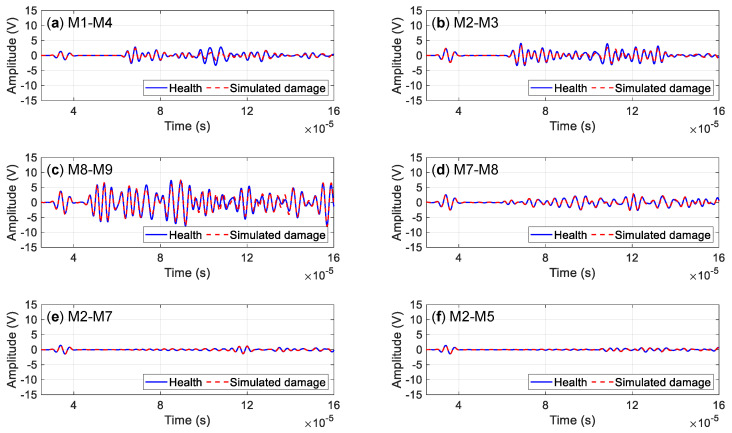
Typical channel signal in healthy and damaged states: (**a**) M1–M4; (**b**) M2–M3; (**c**) M8–M9; (**d**) M7–M8; (**e**) M2–M7; (**f**) M2–M5.

Figure 10 illustrates guided wave signals from different channels. The waveform of the guided wave signal between the health state and the damage state is nearly the same. Changes are observed from the amplitude. The phase change of the guided wave signal is not significant.

**Figure 11:**
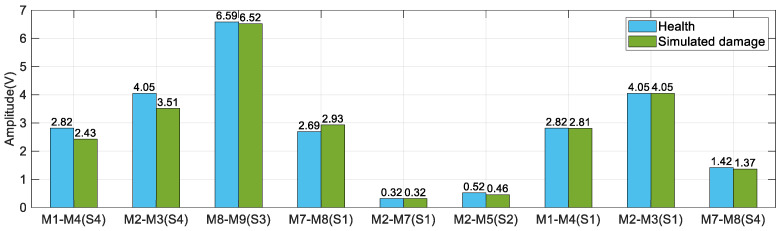
Amplitude changes of typical signal in healthy and damaged states.

Comparing the amplitude changes of guided wave signals in the health state and damage state of each channel, the results are shown in Figure 11. Channels within a single waveguide are more sensitive to damage on the skin. The channel M1–M4 and M2–M3 on the skin is sensitive to simulated damage S4 on the skin. The channel M7–M8 on the stringer joint has a larger amplitude attenuation to the damage S1 in the stringer. The channel on the skin has a poor effect on the simulated damage identification on the stringer joint. The channel passing through three-layer waveguides is insensitive to simulated damage, like the channel M2–M5.

Based on the above results, PZT layout rules for such complex structures are proposed as follows:

1. The signal excited in the skin is difficult to propagate from the skin to the stringer. So, it is better to arrange PZTs on the skin to monitor its damage.
2. When guided waves propagate through a right-angle bend, significant amplitude attenuation and mode conversion occur. Hence, arranging PZTs in the same plane is a preferable option. The propagation of guided waves through the stringer and stringer joint results in signal amplitude increase due to boundary reflections. Therefore, it is worth considering placing channels for interlayer propagation on the stringer and stringer joint, especially when focusing on their connection area. However, guided wave signals propagated through three layers exhibit low amplitude; thus, it is advisable to avoid deploying sensors in such configurations.
3. Monitoring damage through more than two-layer structural elements is challenging. Therefore, it is advisable to avoid using PZT sensors on the skin to monitor damage on the stringer joint or using PZT sensors on the stringer joint to monitor damage on the skin. However, the channels between the stringer and stringer joint benefit from enhanced signal amplitude due to boundary reflections, enabling the guided wave amplitude to perceive the effects of damage.

## 3. GP-Based Probabilistic Mining Diagnosis with Path-Wave Band Feature

This paper proposes the GP-based probabilistic mining diagnosis method for the multi-layer and multi-rivet connection structures with the path-wave band feature, which is implemented in two steps: (1) path-wave band feature extraction of guided waves and (2) GP-based probabilistic mining diagnosis.

### 3.1. Path-Wave Band Feature Extraction of Guided Waves

The guided wave feature, namely the damage index, is extracted to quantify the guided wave signal changes. It is defined by measuring the difference between the monitoring signal and the baseline signal, as shown in Equation (1):

(1) y=h(D(t),H(t)),

where y is the damage index, D(t) is the cracked signal, H(t) is the baseline signal obtained when the structure is in its pristine state, t is the time, and h(⋅) is a damage index function.

There are different kinds of damage indexes in the SHM field [32]. As mentioned above, the propagation path of guided waves is obstructed by cracks, leading to phenomena such as reflection and scattering at the crack location, which results in a decrease in the energy of the guided wave signal received by the sensor. Therefore, the scattering energy damage index is used in Equation (2):

(2) $$y=\frac{\int_{t_{\mathrm{start}}}^{t_{\mathrm{end}}}{\left(H(t)-D(t)\right)}^{2}dt}{\int_{t_{\mathrm{start}}}^{t_{\mathrm{end}}}{\left(H(t)\right)}^{2}dt},$$

where $t_{\mathrm{start}}$ and $t_{\mathrm{end}}$ are the start and end of the time window intercepting the healthy and damaged guided wave signal H(t) and D(t) for evaluating the damage index. This damage index characterizes the loss of signal energy. It gradually increases until the monitored signal becomes zero. Usually, the time window is decided with the packet of the A0 mode or S0 mode of the guided wave signal. However, as indicated above, it is difficult to intercept such packets due to reflection, transmission, and mode conversion introduced by complex boundary conditions. Therefore, this paper proposes a feature extraction method using the path-wave band of guided waves. That is, the signal of reflection, scattering in the propagation path of the guided wave can also have the damage information, which cannot be simply discarded.

Firstly, according to the structural characteristics of complex connected structures, the possible main propagation paths of guided waves are analyzed. The path with the shortest propagation distance of guided wave direct wave and the longest propagation path, including the farthest boundary reflection, are analyzed. Then, the propagation time of the longest path and the shortest path is calculated by using the group velocity of A0 mode and S0 mode, as shown in Equations (3) and (4). Taking these two moments as the starting point and the ending point, respectively, the corresponding guided wave signal is intercepted as the path wave band extracted for computing the damage index.

(3) $$t_{\mathrm{start}}=\frac{L_{\min}}{v_{S0}}-\frac{W}{2},$$

(4) $$t_{\mathrm{end}}=\frac{L_{\max}}{v_{A0}}+\frac{W}{2},$$

where W is the width of signal packets in the time domain, $L_{\min}$ is the shortest propagation distance, $L_{\max}$ is the longest propagation distance.

### 3.2. GP-Based Probabilistic Mining Diagnosis Method

Due to the uncertainties in practical engineering, probabilistic modeling should be considered [33]. Therefore, in this paper, the probabilistic guided wave GP model is adopted to describe the relationship between the damage index y and the crack size a. Theoretically, GP defines a probabilistic distribution over the relationship a=f(y) in Equation (5):

(5) $$a=f\left(y\right)\sim GP\left(m\left(y\right),k\left(y_{p},y_{q}\right)\right),$$

where m(y) is the mean function and $k\left(y_{p},y_{q}\right)$ is the covariance function denoting the distance between the input $y_{p}$ and $y_{q}$.

Based on this definition, the probability distribution $p\left(f\left(y_{1}\right),\ldots ,f\left(y_{n}\right)\right)$ satisfies the joint Gaussian distribution in Equation (6):

(6) a~N(m(y),K(y,y)),

where $a={\left[a_{1},\ldots ,a_{n}\right]}^{T}$, and $y={\left[y_{1},\ldots ,y_{n}\right]}^{T}$, N(⋅,⋅) denotes the Gaussian distribution, whose mean is m(y) and covariance is K(y,y). The mean m(y) is a n×1 matrix that defines the mean values, whose elements are calculated by the mean function. Usually, the mean function of this evaluation model is set as m(⋅)=0. K(y,y) is a n×n matrix whose element is the covariance of $a_{p}$ and $a_{q}$, calculated by the covariance function $k\left(x_{p},x_{q}\right)$. The covariance function means the damage indices similar to each other are likely to correspond to similar crack sizes. Choices of covariance functions include the squared exponential, polynomial, and periodic [34]. They have different properties, and the choice of one over the other typically relies on knowledge of the problem researched. In this paper, considering that the extracted features of complex connection structures vary with the crack length, the covariance function is adopted as Equation (7):

(7) $$\begin{array}{cc} k\left(y_{p},y_{q}\right) & =k_{\mathrm{NN}}\left(y_{p},y_{q}\right)+k_{\mathrm{SE}}\left(y_{p},y_{q}\right)+k_{\mathrm{LIN}}\left(y_{p},y_{q}\right)+k_{\sigma }\left(y_{p},y_{q}\right)\\ & =\beta_{1}^{2}\sin^{-1}\left(\frac{y_{p}\Lambda^{-2}y_{q}{}^{T}}{\sqrt{f\left(y_{p}\right)f\left(y_{q}\right)}}\right)+\beta_{3}^{2}\exp\left(\frac{{\left\|y_{p}-y_{q}\right\|}^{2}}{\beta_{4}^{2}}\right)+\beta_{5}^{2}y_{p}y_{q}+\beta_{6}^{2}\delta_{pq} \end{array},$$

where the neural net covariance function $k_{\mathrm{NN}}$ and square exponential covariance function $k_{\mathrm{SE}}$ have good approximation properties. The linear covariance function $k_{\mathrm{LIN}}$ can describe trends that increase and decrease globally. The $k_{\sigma }$ describes the uncertainties that existed in the dataset. In the neural net covariance function, $f\left(y\right)=1+y\Lambda^{-2}y$ and $\Lambda =diag\left(\beta_{2}^{2}\right)$. In the noise covariance function, $\delta_{pq}$ is the Delta function denoted in Equation (8):

(8) $$\delta_{pq}=\{\begin{array}{cc} 1, & p=q\\ 0, & p\neq q \end{array},$$

The GP model is a data-driven model, which is constructed based on a training dataset containing n pairs of data. This dataset is obtained from a batch of the same structures, denoted in Equation (9):

(9) $${\mathrm{D}}^{\prime }=\left\{\left({y^{\prime }}_{i},{a^{\prime }}_{i}\right)|{}_{i=1}^{n^{\prime }}\right\}=\left(y^{\prime },a^{\prime }\right),$$

where $a^{\prime }$ is the collection of crack size ${a^{\prime }}_{i}$. $y^{\prime }$ is the collection of damage index ${y^{\prime }}_{i}$. $n^{\prime }$ is the number of this training data.

For any damage index $y^{*}$, the joint probability distribution of its corresponding crack size $a^{*}$ and the training data $a^{\prime }$ also follow the joint Gaussian distribution, as shown in Equation (10):

(10) $$\left[\begin{array}{c} a^{\prime }\\ a^{*} \end{array}\right]\sim N\left(\left[\begin{array}{c} m\left(y^{\prime }\right)\\ m\left(y^{*}\right) \end{array}\right],\left[\begin{array}{cc} K\left(y^{\prime },y^{\prime }\right) & K\left(y^{\prime },y^{*}\right)\\ K\left(y^{*},y^{\prime }\right) & k\left(y^{*},y^{*}\right) \end{array}\right]\right),$$

where $K\left(y^{\prime },y^{\prime }\right)$ is a $n^{\prime }\times n^{\prime }$ matrix, $K\left(y^{\prime },y^{*}\right)=K{\left(y^{*},y^{\prime }\right)}^{T}$ is a $n^{\prime }\times 1$ matrix, and $k\left(y^{*},y^{*}\right)$ is a scalar.

For GP model training from the dataset ${\mathrm{D}}^{\prime }$, the hyper-parameters $\beta =\left\{\beta_{1},\beta_{2},\beta_{3},\beta_{4},\beta_{5},\beta_{6}\right\}$ are optimized by minimizing the negative marginal log-likelihood [35] in Equation (11). This is performed by the conjugate gradient algorithm [36].

(11) $$L\left(\beta \right)=-\frac{1}{2}{\left(a^{\prime }\right)}^{T}K{\left(y^{\prime },y^{\prime }\right)}^{-1}a^{\prime }-\frac{1}{2}\log\left|K\left(y^{\prime },y^{\prime }\right)\right|-\frac{n^{\prime }}{2}\log2\pi ,$$

After training, the guided wave-GP crack evaluation model is established and can be used later online. During service, successively obtained damage index from guided wave signal and is substituted into this model to obtain crack size. The mean and variance of the conditional probability distribution of $a_{k}$ corresponding to $y_{k}$ can be deduced by Equation (12) and Equation (13), respectively,

(12) $${\bar{a}}_{k}=K\left(y_{k},y^{\prime }\right){\left[K\left(y^{\prime },y^{\prime }\right)\right]}^{-1}a^{\prime },$$

(13) $$\mathrm{cov}\left(a_{k}\right)=k\left(y_{k}.y_{k}\right)-K\left(y_{k},y^{\prime }\right){\left[K\left(y^{\prime },y^{\prime }\right)\right]}^{-1}K\left(y^{\prime },y_{k}\right),$$

where ${\bar{a}}_{k}$ is the mean of this conditional probability distribution, which is the evaluated crack size with the input $y_{k}$, and $\mathrm{cov}\left(a_{k}\right)$ is the variance by using this model.

## 4. Experimental Validation on Crack Evaluation in MLSSJ Structure

With the sensor layout rules and the crack diagnosis method mentioned above, the following investigates the probabilistic crack evaluation in the complex MLSSJ structure. Here, the crack damage in the stringer joint is addressed since it is more complex than that in the skin.

### 4.1. Setup of Crack Damages in the MLSSJ Structure

**Figure 12:**
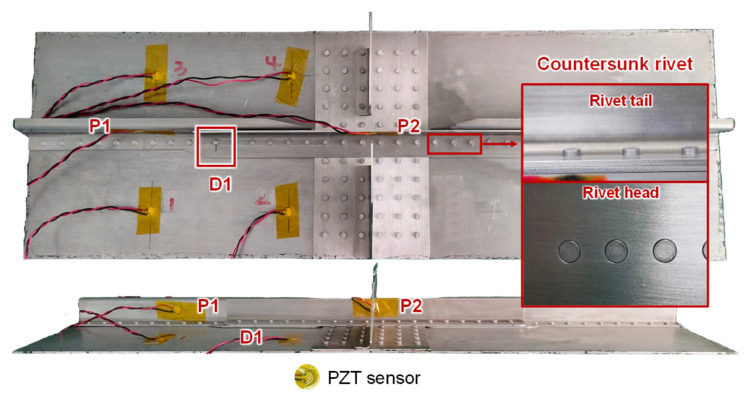
Sensor arrangement and setup for crack monitoring.

**Figure 13:**
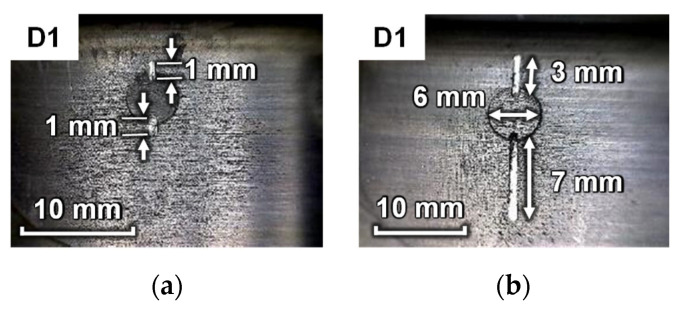
Photographs of machined cracks: (**a**) crack D1 of 2 mm; (**b**) crack D1 of 10 mm.

As shown in Figure 12, the riveted MLSSJ structure is adopted for validation. Countersunk rivets are used to connect these parts. According to experience, the first rivet connecting the stringer and stringer joint is more prone to be cracked. Therefore, real cracks are machined in the stringer joint shown in Figure 13, denoted as crack “D1”. The crack is machined with the computer numerical control (CNC) milling machine. To simulate the actual fatigue crack, the crack is sequentially machined at the edge of the rivet hole. The maximum size of the cracks is 10 mm. Finally, each crack contains 11 states, including the healthy baseline, 1 mm, 2 mm, 3 mm, 4 mm, 5 mm, 6 mm, 7 mm, 8 mm, 9 mm, and 10 mm. Figure 13 shows photographs of machined cracks at lengths of 2 mm and 10 mm. The depth of the crack is controlled so that it only penetrates the stringer joint. The width of the crack is about 0.7 mm.

With the conclusion in Section 2, channel P1–P2 on the stringer and stringer joint is arranged to monitor crack D1, as shown in Figure 12. Once a crack state is machined, guided wave signals are collected from these channels. A total of seven structures labeled as B-1 to B-7 are tested in this experiment. Four sensors on the skin in Figure 12 can be ignored, as they will not be used in the evaluation of the crack D1 in the stringer joint.

### 4.2. Typical Guided Wave Signal in a Batch of MLSSJ Structures

**Figure 14:**
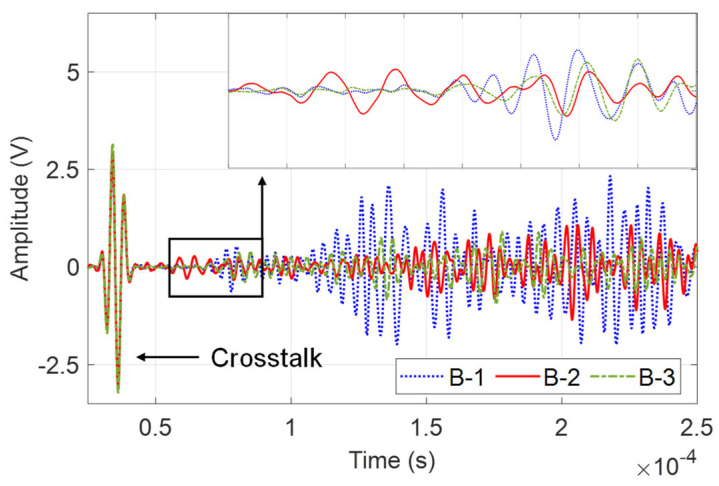
Typical guided wave signals from P1–P2 of different specimens.

The monitoring signals of B-1 to B-7 at the same central frequency are very different. For example, Figure 14 shows typical guided wave signals from channel P1–P2 of specimen B-1, B-2, and B-3, which is excited with the central frequency of 230 kHz. It can be found that their signals differ considerably.

**Figure 15:**
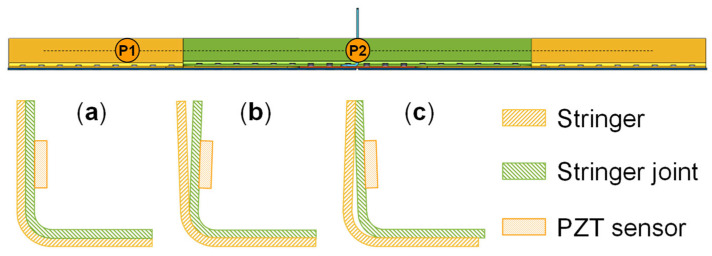
The assembly condition diagram of stringer and stringer joint: (**a**) complete fit; (**b**) top fork fit; (**c**) R-zone gap fit.

**Figure 16:**
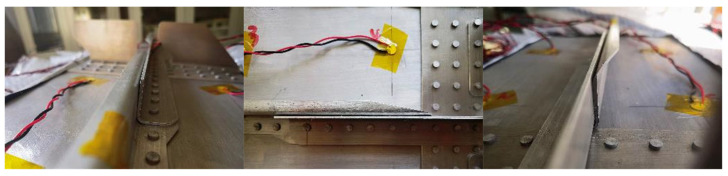
The assembly condition photo of the stringer and stringer joint.

This discrepancy is attributed to the complex contacts between components in the MLSSJ structure. For example, the fabrication and assembling of the bending R-zone of the stringer and the stringer joint differ among different specimens. Figure 15 illustrates the observed conditions of a single cross-section in specimens B-1 to B-7. However, it is difficult to establish a correlation between these assembly conditions and guided wave signals, since the information about the assembly conditions only can visually be accessed from the end or the top of the structure, as depicted in Figure 16. The cross-section along the excitation-sensing path may vary in the region between P1 and P2. Furthermore, other conditions, such as the contact between the bottom plate of the stringer and the stringer joint and the effects of rivet holes, can also contribute to the complexity. In general, the propagation of guided waves is significantly affected by the differences in boundary conditions among the structural components. For the MLSSJ structure, it is difficult to choose a unified wave packet for the batch of structures B-1 to B-7 for crack evaluation.

### 4.3. Guided Wave Crack Evaluation in the MLSSJ Structures

**Figure 17:**
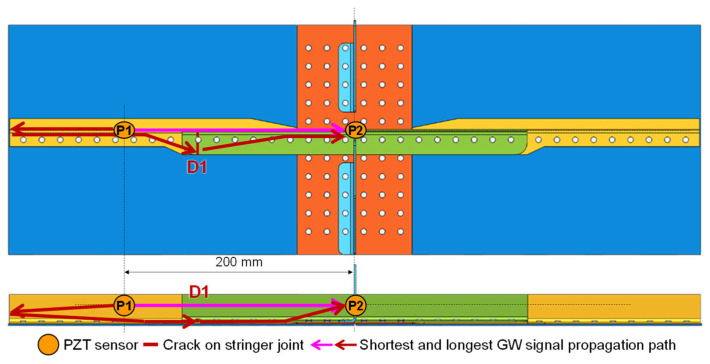
Guided wave signal propagation path for monitoring crack D1.

Then, the guided wave feature, the path-wave band feature extraction of the guided wave, is implemented. Here, the group velocity of the S0 mode is measured as 5294 m/s, and the group velocity of the A0 mode is measured as 2296 m/s in the plate structure with a thickness of 1.5 mm. As shown in Figure 17, the purple arrow represents the shortest direct wave path, and the red arrow represents the longest boundary reflection path of P1–P2 for crack D1. The time window can be calculated by Equations (3) and (4), as the propagating distance is known.

**Figure 18:**
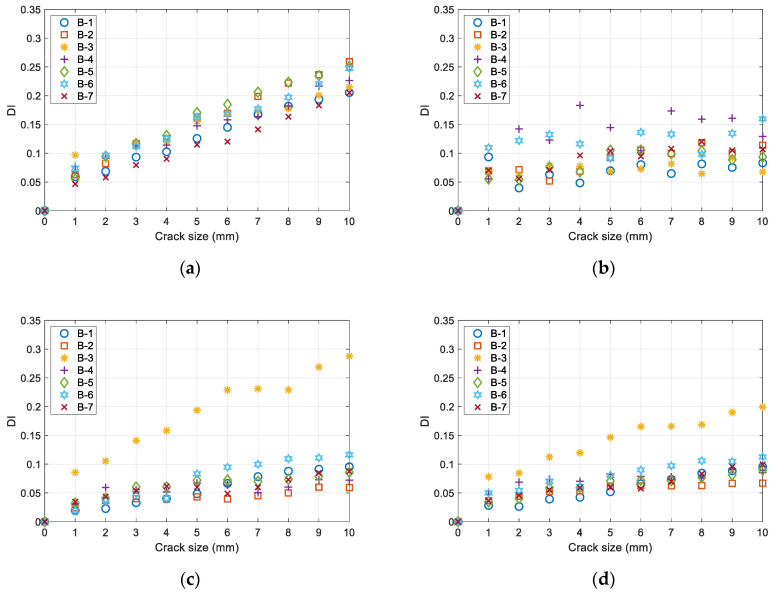
Damage index of P1–P2 extracted with different time windows: (**a**) time window determined by the path-wave band; (**b**) time window determined by S0; (**c**) time window determined by A0; (**d**) time window determined by S0 and A0.

The results of the damage factor calculations for seven specimens with crack lengths ranging from 1 to 10 mm are depicted in Figure 16. In Figure 18a, the time window $\left[t_{\mathrm{start}},t_{\mathrm{end}}\right]$ is evaluated as 7.2 × 10^−5^ to 2.4 × 10^−4^ s, with the corresponding propagation path exemplified in Figure 17. In Figure 18b, the time window is evaluated as 7.2 × 10^−5^ to 8.5 × 10^−5^ s according to the propagation of the S0 mode. In Figure 18c, the time window is evaluated as 1.14 × 10^−4^ to 1.27 × 10^−4^ s according to the propagation of the A0 mode. In Figure 18d, the time window is evaluated as 7.2 × 10^−5^ to 1.27 × 10^−4^ s according to the propagation of the S0 mode and A0 mode.

The damage index in Figure 18a shows a good adherence to linearity. Contrary to this, the damage index in Figure 18b demonstrates a minimal linear trend. This phenomenon could potentially be ascribed to the constrained multi-layer propagation capabilities of the S0 mode. The damage index in Figure 18c,d exhibits a more pronounced linear trend. However, the variation of the damage index with respect to crack size is relatively small, making it challenging to distinguish between different crack sizes. Additionally, in Figure 18c,d, specimen B-3 demonstrates a notably larger damage index compared to the other specimens, leading to a diminished overall consistency in the results among different specimens. In general, the damage index in Figure 18a is superior, not only displaying a higher degree of linearity but also demonstrating better consistency across different specimens.

**Figure 19:**
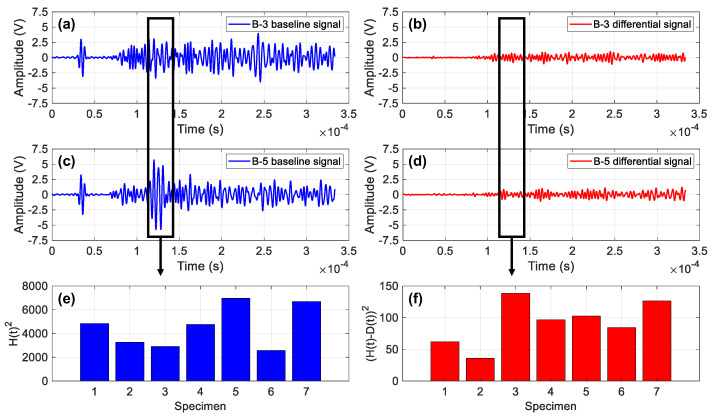
Comparison of damage index between B-3 and B-5: (**a**) baseline signal of B-3; (**b**) differential signal between baseline and 10 mm crack state of B-3; (**c**) baseline signal of B-5; (**d**) differential signal between baseline and 10 mm crack state of B-5; (**e**) quadratic sum of baseline signal with A0 time window; (**f**) quadratic sum of differential signal with A0 time window.

It is evident that the DIs of the B-3 specimen are the largest compared with the rest specimens in Figure 18c,d. This is due to the signal characteristics of specimen B-3. For example, the baseline signals for specimens B-3 and B-5 in their healthy state are shown in Figure 19a,c. Moreover, the differential signals between the baseline and 10 mm crack state are shown in Figure 19b,d. It can be observed that, within the A0 time window, the baseline signal of specimen B-3 is significantly smaller than that of B-5. However, the differential signal of the specimen B-3 is larger than that of B-5. The damage index is the ratio of the two values as shown in Equation (2). Therefore, the DIs of the B-3 are larger than the other specimens in Figure 18c,d, in which the A0 mode is the main signal segment used for calculating the DI.

With the method in Section 3.2, quantitative evaluation of the crack size in the MLSSJ structure is implemented under the leave-one-out framework. That is, a specimen in the seven specimens B-1 to B-7 is used as the target structure for testing. The data from the remaining six specimens are used for constructing the guided wave-GP diagnosis model. For example, if specimen B-1 is used as the target structure, the data from specimens B-2, B-3, B-4, B-5, B-6, and B-7 are used for training the GP model. If specimen B-2 is used as the target structure, the data from specimens B-1, B-3, B-4, B-5, B-6, and B-7 are used for training the GP model.

**Figure 20:**
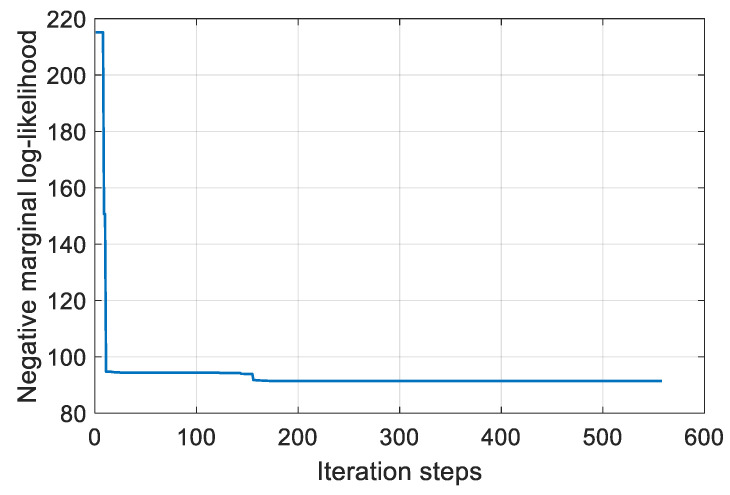
The negative marginal log-likelihood of specimen B-1.

Taking specimen B-1 as the target structure, training data from the remaining six specimens are obtained as ${\mathrm{D}}^{\prime }=\left\{\left({y^{\prime }}_{i},{a^{\prime }}_{i}\right)|{}_{i=1}^{6}\right\}$, where $a^{\prime }$ is the crack length and ${y^{\prime }}_{i}$ is the damage index evaluated with the guided wave signal collected at the crack length $a^{\prime }$. Then, the training process of the GP model uses the conjugate gradient method [36] to find the model hyperparameters $\beta =\left\{\beta_{1},\beta_{2},\beta_{3},\beta_{4},\beta_{5},\beta_{6}\right\}$ that minimize the negative log-likelihood values of the training data ${\mathrm{D}}^{\prime }$, as shown in Equation (11). The training is performed with random initial model hyperparameters and iterates these hyperparameter values with the conjugate gradient method. Figure 20 illustrates the variation of the negative marginal log-likelihood with each iteration when specimen B-1 is considered as the target structure for crack assessment. It can be observed that after hundreds of iterations, the model hyperparameters converge to optimal ones.

**Figure 21:**
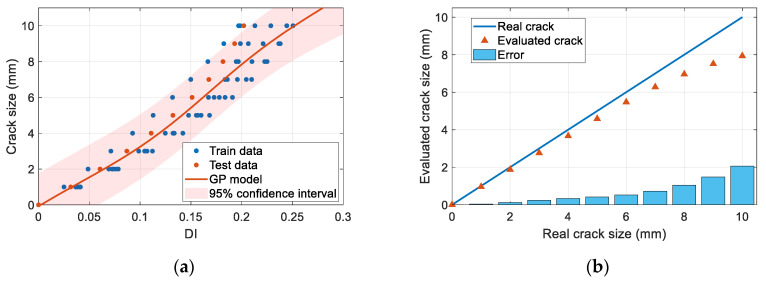
Quantitative damage evaluation results of B-1 with GW-Gaussian process: (**a**) Gaussian process calibration models; (**b**) quantitative assessment of the crack.

Figure 21a illustrates the case when specimen B-1 is used as the target structure. Figure 21b gives the evaluated crack size of specimen B-1, in which the absolute errors are evaluated. In addition, with the B-1 to B-7 as the target structure, respectively, the evaluation error of the crack size on the stringer is obtained in Table 1 under the leave-one-out framework.

In Figure 21b, the error of specimen B-1 increases with the crack size. This is an occasional phenomenon since the results of specimens B-2, B-3, B-4, B-5, and B-6 do not have this trend. The errors of specimen B-2, B-3, B-4, B-5, B-6 are irregular. Due to the inherent uncertainties during structure manufacturing, crack geometries, and assembling, guided wave signals in the batch of complex multi-layer and multi-rivet connection structures are quite different. Therefore, the relationship of the damage index versus the crack length is dispersive, as shown in Figure 18a. The data of B-1 are located at the edge of the data distribution, and the GP model represents the mean trend of the data. The trained GP model inevitably has errors. This error cannot be eliminated at the offline stage, since specimen B-1 is assumed to be unknown. To reduce the dispersion of the crack evaluation, on-line updating strategies for the GP model can be developed. That is, when specimen B-1 is in service, inspection data about the real crack can be used to correct this bias, reducing the evaluation error.

From Table 1, most of the evaluation error of the crack size is below 2 mm. The root mean squared error in Table 1 is 1.0 mm. The average evaluation error of the seven pieces is the smallest at 1 mm, the average evaluation error is 0.2 mm, and the maximum average error is 1.3 mm at 10 mm. Among the seven specimens, the average evaluation error of B-4 under each crack size is the smallest, which is 0.5 mm, and the average evaluation error of B-7 is the largest, which is 1.5 mm. In general, the quantitative crack assessment results prove the effectiveness of the proposed probabilistic diagnosis method in Section 3.

## 5. Conclusions

This paper focuses on the crack damage probabilistic diagnosis using guided waves for complex connection structures in civil aircraft fuselage. The research is conducted on complex multi-layer stringer splice joint structures located on the skin of the aircraft fuselage frame section. The MLSSJ structure is designed with the 1:1 ratio of the key region of real structures. It is a truncation of the real structure, with the skin and the stringer being truncated compared to a complete structure. This truncation may introduce additional reflections of the guided waves compared to the real structure. In a larger section of the fuselage, parallel stringers and joints also introduce reflections. Thus, the dimensions of the structure are designed with reference to the spacing between two stringers. Moreover, a wave-absorbing material is applied around the structure during experiments, which can reduce the energy of the reflection introduced by truncation, so that simulating the situation in a larger section of the fuselage. Therefore, the segment and results of this paper can be related to a larger section of the fuselage, i.e., the practical engineering applications.

The paper investigates the guided wave propagation characteristics under the influence of complex structural connections, analyzing the changes in signal amplitude and phase as well as the mode transitions in the MLSSJ structure. The research also examines the impact of guided wave damage scattering signals after passing through multiple rivet holes or multi-layer structures on the damage identification capability. Guided wave sensor layout rules for damage monitoring in complex multi-layer connection structures of commercial aircraft are proposed. The paper explores a crack damage probabilistic diagnosis method for complex multi-layer stringer joint structures based on the scattering signals, discusses the probability of detection for damage detection based on different guided wave feature extraction methods, and presents a quantitative evaluation method for crack damage in complex MLSSJ structures of civil aircraft using GW-GP probabilistic diagnosis method. The effectiveness of this method is validated through experimental results involving actual cracks, confirming the validity of the proposed approach.

## Figures and Tables

**Table 1:** Quantitatively evaluation error of crack D1.

Specimens	Quantitatively Evaluation Error (mm)									
	1 mm	2 mm	3 mm	4 mm	5 mm	6 mm	7 mm	8 mm	9 mm	10 mm
B-1	0.0	0.1	0.2	0.3	0.4	0.5	0.7	1.0	1.5	2.1
B-2	0.2	0.4	0.5	0.8	1.2	1.4	1.4	1.2	0.7	0.2
B-3	0.3	0.5	0.7	0.8	0.9	0.7	0.3	0.3	1.2	2.2
B-4	0.2	0.2	0.3	0.4	0.5	0.4	0.2	0.2	0.8	1.5
B-5	0.3	0.6	0.8	1.2	1.6	1.8	1.7	1.3	0.7	0.1
B-6	0.3	0.4	0.6	0.7	1.0	1.1	0.9	0.5	0.1	0.9
B-7	0.3	0.6	0.9	1.3	1.6	1.8	1.9	1.9	2.0	2.2

## Data Availability

Data are contained within the article.
